# Hydrogen-bonding and π–π stacking inter­actions in tris­(1,10-phenanthroline-κ^2^ *N*,*N*′)nickel(II) bis­{[1-*tert*-butyl­imidazole-2(3*H*)-thione-κ*S*]trichloridonickelate(II)} acetonitrile disolvate

**DOI:** 10.1107/S1600536808018060

**Published:** 2008-06-19

**Authors:** Udai P. Singh, Vaibhave Aggarwal

**Affiliations:** a Department of Chemistry, Indian Institute of Technology Roorkee, Roorkee 247667, India

## Abstract

The asymmetric unit of the title complex, [Ni(C_12_H_8_N_2_)_3_][NiCl_3_(C_7_H_12_N_2_S)]_2_·2CH_3_CN, consists of one anion, one-half of a cation and one acetonitrile mol­ecule. The Ni^II^ atom in the [Ni(phen)_3_]^2+^ cation (phen is 1,10-phenanthroline) lies on an inversion centre in an octa­hedral environment, whereas in the [NiCl_3_(tm)]^−^ anion [tm is 1-*tert*-butyl­imidazole-2(3*H*)-thione], the geometry is distorted tetra­hedral. In the crystal structure, inter­molecular C—H⋯Cl hydrogen bonds and π–π stacking inter­actions (centroid–centroid distance = 3.52 Å) lead to the formation of a three-dimensional framework. One of the methyl groups of the *tert*-butyl group of *N*-*tert*-butyl-2-thio­imidazole is disordered between two equally populated positions.

## Related literature

For general background, see: Fatimi *et al.* (1994 ▶); Iradyan *et al.* (1987 ▶); Suescun *et al.* (1999 ▶); Yu *et al.* (2003 ▶); Fang & Dai (2006 ▶); Chen *et al.*, (2007 ▶); Senda *et al.* (2006 ▶). For synthesis details, see: Kister *et al.* (1979 ▶).
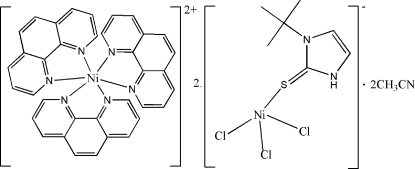


## Experimental

### 

#### Crystal data

[Ni(C_12_H_8_N_2_)_3_][NiCl_3_(C_7_H_12_N_2_S)]_2_·2C_2_H_3_N
*M* *_r_* = 1324.04
Monoclinic, 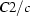

*a* = 22.8953 (15) Å
*b* = 15.2934 (10) Å
*c* = 19.9417 (19) Å
β = 123.543 (3)°
*V* = 5819.7 (8) Å^3^
*Z* = 4
Mo *K*α radiation
μ = 1.36 mm^−1^
*T* = 298 (2) K
0.24 × 0.20 × 0.18 mm

#### Data collection

Bruker Kappa APEXII CCD area-detector diffractometer
Absorption correction: multi-scan (*SADABS*; Sheldrick, 1996 ▶) *T*_min_ = 0.737, *T*_max_ = 0.792
30921 measured reflections
5178 independent reflections
3346 reflections with *I* > 2σ(*I*)
*R*_int_ = 0.076

#### Refinement

*R*[*F* ^2^ > 2σ(*F* ^2^)] = 0.050
*wR*(*F* ^2^) = 0.157
*S* = 1.07
5178 reflections
370 parameters
3 restraints
H atoms treated by a mixture of independent and constrained refinement
Δρ_max_ = 1.24 e Å^−3^
Δρ_min_ = −0.55 e Å^−3^

### 

Data collection: *APEX2* (Bruker, 2007 ▶); cell refinement: *SAINT* (Bruker, 2007 ▶); data reduction: *SAINT*; program(s) used to solve structure: *SHELXS97* (Sheldrick, 2008 ▶); program(s) used to refine structure: *SHELXL97* (Sheldrick, 2008 ▶); molecular graphics: *SHELXTL* (Sheldrick, 2008 ▶) and *DIAMOND* (Brandenburg, 1999 ▶); software used to prepare material for publication: *SHELXL97*.

## Supplementary Material

Crystal structure: contains datablocks I, global. DOI: 10.1107/S1600536808018060/su2050sup1.cif

Structure factors: contains datablocks I. DOI: 10.1107/S1600536808018060/su2050Isup2.hkl

Additional supplementary materials:  crystallographic information; 3D view; checkCIF report

## Figures and Tables

**Table 1:** Hydrogen-bond geometry (Å, °)

*D*—H⋯*A*	*D*—H	H⋯*A*	*D*⋯*A*	*D*—H⋯*A*
N4—H4⋯Cl2	0.85 (6)	2.37 (7)	3.178 (6)	160 (6)
C2—H2⋯Cl3^i^	0.82 (7)	2.77 (7)	3.552 (8)	160 (4)
C5—H5*C*⋯S1	0.96	2.75	3.402 (9)	126
C7—H7*A*⋯S1	0.96	2.68	3.409 (8)	133
C10—H10⋯Cl3^ii^	0.93	2.72	3.557 (7)	151
C25—H25⋯N6^iii^	0.93	2.60	3.502 (9)	162

Symmetry codes: (i) 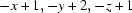; (ii) 
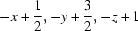
; (iii) 
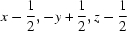
.

**Fig. 1.**
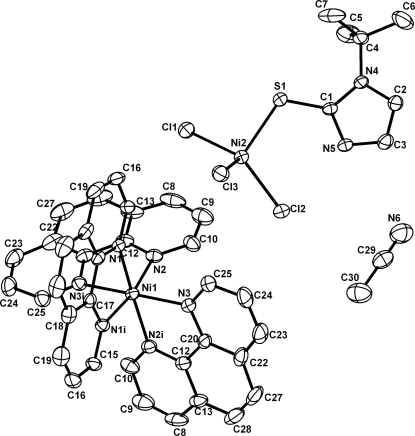
The molecular structure of the compound (I), shown with 30% probability displacement ellipsoids [H atoms have been omitted for clarity; Symmetry code: (i) -x, y, 0.5 - z.]

**Fig. 2.**
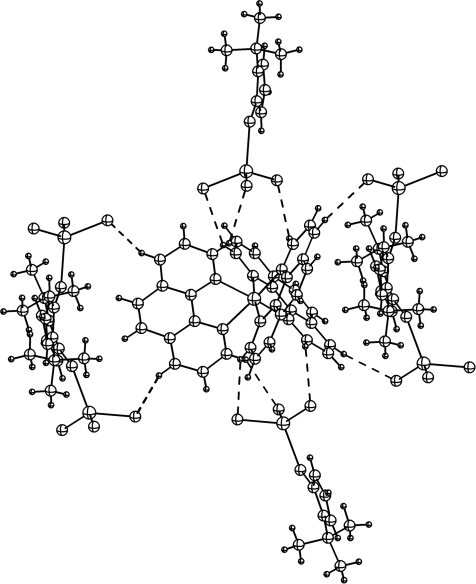
A view of the intermolecular C—H···Cl interactions involving the [Ni(phen)3]+ cation.

**Fig. 3.**
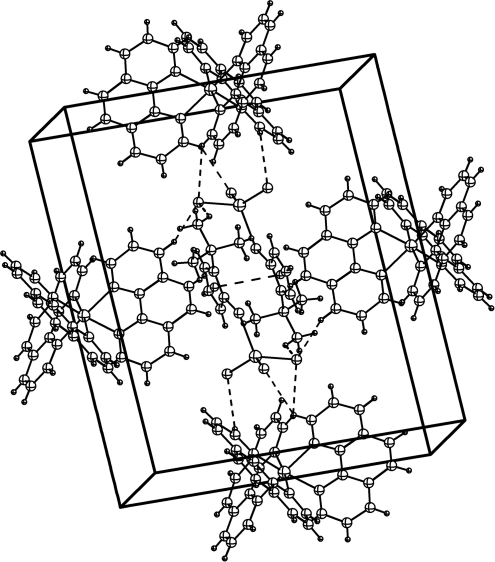
A view of the intermolecular π–π stacking and C—H···Cl interactions involving the [Ni(tm)(Cl)3]- anion.

**Fig. 4.**
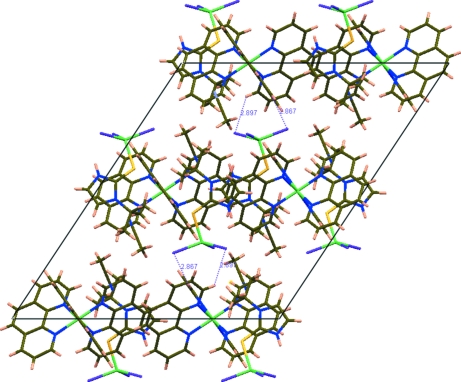
A view along the b axis of the crystal packing of complex (I), showing the interaction between two layers.

## supplementary crystallographic information

### Comment

2-Thioimidazole (N,*N*,*S* donors) and its alkyl derivatives have antithyroid activity and platelet inhibitory effects. (Fatimi *et al.*, 1994; Iradyan *et al.*, 1987). The literature revealed the presence of various nickel(II) complexes with phenanthroline (Suescun *et al.*, 1999; Yu *et al.* (2003); Fang & Dai, 2006; Chen *et al.*, 2007) but none with *N*-*tert*-butyl-2-thioimidazole, except the one reported recently (Senda *et al.*, 2006). The present work reports the first structure of a nickel(II) complex with *N*-*tert*-butyl-2-thioimidazole and 1,10 phenanthroline, containing a tetrahedral anion, [Ni(tm)(Cl)_3_]^-^, and an octahedral cation, [Ni(phen)_3_]^2+^.

The title complex (I) is centrosymmetric, ionic in nature and comprises of one complex cation [Ni(phen)_3_]^2+^ and two complex anions [Ni(tm)(Cl)_3_]^-^ in the unit cell (Fig. 1). The metal centre in [Ni(phen)_3_]^2+^ is in an octahedral environment, the equatorial plane of which is formed by four phen nitrogen atoms, and the axial positions are occupied by another two nitrogen atoms of phen, with Ni1—N bond distances in the range of 2.079 (4)–2.100 (4) Å. The dihedral angles between the meanplanes of the neighboring phen rings are 77.53°, 86.07° and 85.97°. The *cis*-angles in the octahedron deviate only slightly from 90° and the *trans* angle in the axial position is almost linear *i.e.* 170.54 (15)°. The nickel(II) atom in the [Ni(tm)(Cl)_3_]^-^ anion is coordinated by three chlorine atoms and one sulfur atom of tm in a distorted tetrahedral geometry. The Ni—Cl bond distances are in the range of 2.251 (15) to 2.275 (15) Å. In the anion the two short bond distances (Ni2—Cl3 and Ni2—Cl1 of 2.2507 (15) and 2.253 (2) Å, respectively) and two long bond distances (Ni2—S1 and Ni2—Cl2 of 2.3054 (17) and 2.2753 (16) Å, respectively) make the geometry distorted tetrahedral.

Due to the presence of several intermolecular interactions between the three chloride ions bonded to atom Ni2 and the hydrogen atoms present on the phen rings, the complex cation is linked to six complex anions (Fig. 2 and Table 1), whereas the complex anions are linked to four complex cations through C—H···Cl hydrogen bonds (Fig. 3 and Table 1). In the crystal packing of complex (I) two layers are linked by hydrogen bonds in the bc plane. The [Ni(phen)_3_]^2+^ cations and the [Ni(tm)(Cl)_3_]^-^ anions interact with each other via hydrogen bonds formed by the terminally coordinated chloride ions of the complex anion (Cl1, Cl2 and Cl3) and the hydrogen atoms present on the phen ligands (Fig. 4). The two complex anions also interact with one another through π–π stacking, with a separation of ca. 3.52 Å, and intermolecular C—H···Cl and  C—H···N interactions  involving the hydrogen atom of the middle ring of phenanthroline and the nitrogen atom of the acetonitrile molecule present in the lattice (Table 1).

### Experimental

All the reagents were of commercial grade and were used as received. N-tert-butyl-thioimidazole (tm) was synthesized by a literature method (Kister *et al.*, 1979). To a solution of NiCl_2_.6H_2_O (1.0 mmol) in 5 ml methanol, a methanolic solution of N-tert-butyl-2-thioimidazole (1.0 mmol) was added and the mixture stirred for 40 minutes. This mixture was then added to the solution obtained by mixing NiCl_2_.6H_2_O (1.0 mmol) in methanol with a methanolic solution of 1,10-phenanthroline (3.0 mmol). The whole reaction mixture was stirred for a further 30 minutes. The clear solution obtained was filtered and evaporated to dryness. The solid compound obtained was dissolved in acetonitrile and green single crystals, suitable for X-ray analysis, were obtained by slow evaporation at room temperature. Yield 62%. Analysis calculated for C_50_H_48_N_10_S_2_Cl_6_Ni_3_: C 48.30, H 3.86, N 11.27, S 5.15%; found: C 48.24, H 3.78, N 11.21, S 5.12%. Selected IR frequencies (KBr, ν, cm^-1^): 725 (s), 849 (s), 1369 (w), 1575 (m), 1623 (w), 3060 (w), 3412 (s).

### Refinement

One of the methyl groups of the *tert*-butyl group of N-*tert*-butyl-2-thioimidazole is disordered between two equally populated positions (C6 and C6A; H6A and H6A1; H6B and H6B1; H6C and H6C1). C-bound H atoms were placed in geometrically idealized positions, with Csp2—H = 0.93 Å and Csp3—H = 0.96 Å, and treated as riding atoms with *U*_iso_(H) = 1.2Ueq(C). H atoms attached to the O atoms were located in a difference Fourier map and refined as riding in their as found positions, with *U*_iso_(H) = 1.5Ueq(O).

### Crystal data

**Table:**

[Ni(C_12_H_8_N_2_)_3_][NiCl_3_(C_7_H_12_N_2_S_1_)]_2_·2C_2_H_3_N	*F*_000_ = 2720
*M**_r_* = 1324.04	*D*_x_ = 1.511 Mg m^−^^3^
Monoclinic, *C*2/*c*	Mo *K*α radiation λ = 0.71073 Å
Hall symbol: -C 2yc	Cell parameters from 5736 reflections
*a* = 22.8953 (15) Å	θ = 1.7–25.1º
*b* = 15.2934 (10) Å	µ = 1.36 mm^−^^1^
*c* = 19.9417 (19) Å	*T* = 298 (2) K
β = 123.543 (3)º	Block, green
*V* = 5819.7 (8) Å^3^	0.24 × 0.20 × 0.18 mm
*Z* = 4	

### Data collection

**Table:**

Bruker Kappa APEXII CCD area-detector diffractometer	5178 independent reflections
Radiation source: fine-focus sealed tube	3346 reflections with *I* > 2σ(*I*)
Monochromator: graphite	*R*_int_ = 0.076
*T* = 298(2) K	θ_max_ = 25.1º
φ and ω scans	θ_min_ = 1.7º
Absorption correction: multi-scan(SADABS; Sheldrick, 1996)	*h* = −26→26
*T*_min_ = 0.737, *T*_max_ = 0.792	*k* = −17→17
30921 measured reflections	*l* = −22→22

### Refinement

**Table:**

Refinement on *F*^2^	Secondary atom site location: difference Fourier map
Least-squares matrix: full	Hydrogen site location: inferred from neighbouring sites
*R*[*F*^2^ > 2σ(*F*^2^)] = 0.050	H atoms treated by a mixture of independent and constrained refinement
*wR*(*F*^2^) = 0.157	*w* = 1/[σ^2^(*F*_o_^2^) + (0.0987*P*)^2^ + 2.7947*P*] where *P* = (*F*_o_^2^ + 2*F*_c_^2^)/3
*S* = 1.07	(Δ/σ)_max_ < 0.001
5178 reflections	Δρ_max_ = 1.24 e Å^−^^3^
370 parameters	Δρ_min_ = −0.55 e Å^−^^3^
3 restraints	Extinction correction: none
Primary atom site location: structure-invariant direct methods	

### Special details

**Table:**

Geometry. Bond distances, angles etc. have been calculated using the rounded fractional coordinates. All su's are estimated from the variances of the (full) variance-covariance matrix. The cell esds are taken into account in the estimation of distances, angles and torsion angles

### Fractional atomic coordinates and isotropic or equivalent isotropic displacement parameters (Å^2^)

**Table:**

	*x*	*y*	*z*	*U*_iso_*/*U*_eq_	Occ. (<1)
Ni1	0.00000	0.00042 (5)	0.25000	0.0324 (3)	
N1	0.0571 (2)	0.1054 (2)	0.3259 (2)	0.0353 (12)	
N2	0.0560 (2)	−0.0924 (2)	0.3397 (2)	0.0402 (12)	
N3	0.0611 (2)	−0.0069 (3)	0.2013 (2)	0.0394 (12)	
C8	0.1142 (3)	0.1047 (4)	0.3998 (3)	0.0432 (17)	
C9	0.1465 (3)	0.1812 (4)	0.4422 (3)	0.0543 (19)	
C10	0.1193 (3)	0.2601 (4)	0.4082 (4)	0.060 (2)	
C11	0.0597 (3)	0.2640 (3)	0.3294 (3)	0.048 (2)	
C12	0.0304 (2)	0.1842 (3)	0.2905 (3)	0.0359 (17)	
C13	0.0280 (3)	0.3432 (4)	0.2882 (4)	0.065 (3)	
C14	0.1128 (3)	−0.1368 (3)	0.3578 (3)	0.0534 (19)	
C15	0.1476 (4)	−0.1940 (4)	0.4220 (4)	0.069 (2)	
C16	0.1245 (4)	−0.2030 (4)	0.4708 (4)	0.071 (2)	
C17	0.0649 (3)	−0.1584 (3)	0.4557 (3)	0.0542 (19)	
C18	0.0313 (3)	−0.1036 (3)	0.3870 (3)	0.0424 (17)	
C19	0.1208 (3)	0.0335 (4)	0.2245 (3)	0.0503 (17)	
C20	0.1528 (3)	0.0259 (4)	0.1814 (4)	0.066 (3)	
C21	0.1223 (4)	−0.0231 (5)	0.1145 (4)	0.068 (3)	
C22	0.0604 (3)	−0.0683 (4)	0.0887 (4)	0.058 (2)	
C23	0.0315 (3)	−0.0582 (3)	0.1348 (3)	0.0410 (17)	
C24	0.0231 (4)	−0.1241 (5)	0.0183 (4)	0.072 (3)	
C25	−0.0359 (4)	−0.1657 (4)	−0.0022 (4)	0.071 (3)	
Ni2	0.29979 (3)	0.96924 (4)	0.47969 (4)	0.0434 (2)	
Cl1	0.25801 (8)	0.97552 (11)	0.55872 (9)	0.0625 (6)	
Cl2	0.27465 (7)	0.88260 (10)	0.37416 (8)	0.0583 (5)	
Cl3	0.27772 (7)	1.10207 (9)	0.42193 (8)	0.0528 (5)	
S1	0.41282 (7)	0.93790 (9)	0.58439 (8)	0.0452 (4)	
N4	0.4404 (2)	0.8996 (3)	0.4710 (3)	0.0408 (16)	
N5	0.5332 (2)	0.8910 (3)	0.5920 (2)	0.0381 (12)	
C1	0.4636 (3)	0.9081 (3)	0.5493 (3)	0.0359 (17)	
C2	0.5514 (3)	0.8728 (4)	0.5376 (3)	0.0479 (19)	
C3	0.4945 (3)	0.8778 (4)	0.4641 (3)	0.0481 (17)	
C4	0.5832 (3)	0.8870 (4)	0.6823 (3)	0.0452 (17)	
C5	0.5869 (4)	0.9750 (4)	0.7181 (4)	0.089 (3)	
C6	0.6584 (6)	0.8705 (16)	0.7056 (9)	0.093 (6)	0.75 (3)
C7	0.5581 (4)	0.8178 (5)	0.7141 (4)	0.077 (3)	
C6A	0.643 (2)	0.830 (4)	0.695 (3)	0.093 (6)	0.25 (3)
N6	0.3858 (4)	0.7541 (4)	0.3045 (4)	0.091 (3)	
C26	0.3366 (4)	0.7841 (4)	0.2533 (4)	0.066 (3)	
C27	0.2734 (4)	0.8224 (5)	0.1875 (5)	0.093 (3)	
H8	0.13350	0.05120	0.42420	0.0520*	
H9	0.18670	0.17810	0.49400	0.0650*	
H10	0.14000	0.31130	0.43680	0.0720*	
H13	0.04640	0.39620	0.31450	0.0780*	
H14	0.13000	−0.12900	0.32550	0.0640*	
H15	0.18620	−0.22550	0.43130	0.0830*	
H16	0.14870	−0.23950	0.51540	0.0860*	
H19	0.14210	0.06790	0.27070	0.0600*	
H20	0.19480	0.05460	0.19920	0.0790*	
H21	0.14270	−0.02700	0.08520	0.0810*	
H24	0.04090	−0.13130	−0.01350	0.0870*	
H25	−0.05840	−0.20020	−0.04830	0.0850*	
H2	0.593 (3)	0.867 (3)	0.555 (3)	0.036 (14)*	
H3	0.486 (3)	0.868 (4)	0.409 (4)	0.061 (16)*	
H5A	0.59570	1.01920	0.69050	0.1340*	
H5B	0.62410	0.97510	0.77410	0.1340*	
H4	0.397 (3)	0.897 (3)	0.435 (3)	0.039 (15)*	
H6A	0.67440	0.92000	0.69040	0.1380*	0.75 (3)
H6B	0.65930	0.81930	0.67830	0.1380*	0.75 (3)
H6C	0.68860	0.86190	0.76270	0.1380*	0.75 (3)
H7A	0.50960	0.82750	0.69350	0.1160*	
H7B	0.58520	0.82040	0.77180	0.1160*	
H7C	0.56360	0.76130	0.69730	0.1160*	
H5C	0.54330	0.98700	0.71250	0.1340*	
H6A1	0.66990	0.86260	0.67940	0.1380*	0.25 (3)
H6A2	0.62430	0.77870	0.66220	0.1380*	0.25 (3)
H6A3	0.67260	0.81410	0.75030	0.1380*	0.25 (3)
H27A	0.24970	0.85310	0.20790	0.1400*	
H27B	0.24340	0.77720	0.15140	0.1400*	
H27C	0.28470	0.86250	0.15930	0.1400*	

### Atomic displacement parameters (Å^2^)

**Table:**

	*U*^11^	*U*^22^	*U*^33^	*U*^12^	*U*^13^	*U*^23^
Ni1	0.0334 (5)	0.0360 (5)	0.0257 (5)	0.0000	0.0151 (4)	0.0000
N1	0.035 (2)	0.042 (2)	0.028 (2)	0.0003 (18)	0.0168 (19)	−0.0034 (18)
N2	0.042 (2)	0.035 (2)	0.034 (2)	−0.0035 (19)	0.015 (2)	0.0018 (17)
N3	0.039 (2)	0.042 (2)	0.036 (2)	−0.0011 (19)	0.020 (2)	−0.0032 (18)
C8	0.035 (3)	0.060 (3)	0.030 (3)	−0.001 (2)	0.015 (2)	−0.005 (2)
C9	0.046 (3)	0.081 (4)	0.034 (3)	−0.016 (3)	0.021 (3)	−0.019 (3)
C10	0.077 (4)	0.062 (4)	0.055 (4)	−0.023 (3)	0.045 (4)	−0.029 (3)
C11	0.062 (4)	0.046 (3)	0.051 (4)	−0.010 (3)	0.041 (3)	−0.014 (3)
C12	0.043 (3)	0.040 (3)	0.035 (3)	−0.003 (2)	0.028 (2)	−0.004 (2)
C13	0.092 (5)	0.039 (3)	0.087 (5)	−0.016 (3)	0.064 (4)	−0.015 (3)
C14	0.042 (3)	0.043 (3)	0.057 (4)	0.007 (3)	0.016 (3)	−0.001 (3)
C15	0.062 (4)	0.050 (4)	0.063 (4)	0.008 (3)	0.014 (4)	0.007 (3)
C16	0.069 (4)	0.043 (3)	0.046 (4)	0.000 (3)	−0.003 (3)	0.014 (3)
C17	0.063 (4)	0.039 (3)	0.034 (3)	−0.014 (3)	0.010 (3)	0.005 (2)
C18	0.047 (3)	0.033 (3)	0.035 (3)	−0.009 (2)	0.015 (3)	−0.001 (2)
C19	0.044 (3)	0.057 (3)	0.051 (3)	−0.001 (3)	0.027 (3)	−0.002 (3)
C20	0.053 (4)	0.085 (5)	0.078 (5)	0.008 (3)	0.047 (4)	0.015 (4)
C21	0.074 (5)	0.091 (5)	0.064 (5)	0.021 (4)	0.054 (4)	0.018 (4)
C22	0.077 (4)	0.059 (3)	0.051 (4)	0.034 (3)	0.043 (3)	0.016 (3)
C23	0.049 (3)	0.040 (3)	0.034 (3)	0.014 (2)	0.023 (3)	0.005 (2)
C24	0.105 (6)	0.078 (4)	0.051 (4)	0.027 (4)	0.054 (4)	0.000 (3)
C25	0.104 (6)	0.059 (4)	0.041 (4)	0.022 (4)	0.035 (4)	−0.005 (3)
Ni2	0.0394 (4)	0.0561 (4)	0.0325 (4)	0.0045 (3)	0.0185 (3)	−0.0031 (3)
Cl1	0.0540 (9)	0.0980 (12)	0.0409 (8)	0.0098 (8)	0.0297 (7)	0.0020 (7)
Cl2	0.0496 (8)	0.0748 (10)	0.0400 (8)	0.0050 (7)	0.0181 (7)	−0.0152 (7)
Cl3	0.0476 (8)	0.0590 (8)	0.0495 (9)	−0.0008 (6)	0.0254 (7)	0.0015 (6)
S1	0.0413 (7)	0.0626 (8)	0.0318 (7)	0.0068 (6)	0.0203 (6)	−0.0018 (6)
N4	0.042 (3)	0.053 (3)	0.026 (2)	0.000 (2)	0.018 (2)	−0.0013 (19)
N5	0.039 (2)	0.051 (2)	0.026 (2)	0.0010 (19)	0.019 (2)	0.0021 (18)
C1	0.041 (3)	0.040 (3)	0.027 (3)	−0.001 (2)	0.019 (2)	0.002 (2)
C2	0.039 (3)	0.067 (4)	0.043 (3)	−0.004 (3)	0.026 (3)	−0.003 (3)
C3	0.051 (3)	0.061 (3)	0.040 (3)	−0.003 (3)	0.030 (3)	0.000 (3)
C4	0.040 (3)	0.065 (3)	0.029 (3)	0.004 (3)	0.018 (2)	0.006 (2)
C5	0.096 (6)	0.076 (5)	0.040 (4)	−0.009 (4)	0.003 (4)	−0.008 (3)
C6	0.036 (6)	0.183 (16)	0.043 (6)	0.002 (7)	0.012 (6)	0.004 (9)
C7	0.080 (5)	0.091 (5)	0.050 (4)	−0.001 (4)	0.029 (4)	0.023 (3)
C6A	0.036 (6)	0.183 (16)	0.043 (6)	0.002 (7)	0.012 (6)	0.004 (9)
N6	0.103 (5)	0.105 (5)	0.062 (4)	0.008 (4)	0.043 (4)	0.005 (4)
C26	0.088 (5)	0.066 (4)	0.054 (4)	−0.010 (4)	0.045 (4)	−0.002 (3)
C27	0.099 (6)	0.080 (5)	0.073 (5)	0.004 (4)	0.030 (5)	0.006 (4)

### Geometric parameters (Å, °)

**Table:**

Ni1—N1	2.095 (3)	C21—C22	1.395 (12)
Ni1—N2	2.079 (3)	C22—C24	1.450 (10)
Ni1—N3	2.101 (5)	C22—C23	1.407 (10)
Ni1—N1^i^	2.095 (3)	C24—C25	1.337 (13)
Ni1—N2^i^	2.079 (3)	C8—H8	0.9300
Ni1—N3^i^	2.101 (5)	C9—H9	0.9300
Ni2—Cl2	2.2753 (16)	C10—H10	0.9300
Ni2—Cl3	2.2507 (15)	C13—H13	0.9300
Ni2—S1	2.3054 (17)	C14—H14	0.9300
Ni2—Cl1	2.253 (2)	C15—H15	0.9300
S1—C1	1.717 (7)	C16—H16	0.9300
N1—C8	1.324 (7)	C19—H19	0.9300
N1—C12	1.359 (6)	C20—H20	0.9300
N2—C18	1.352 (8)	C21—H21	0.9300
N2—C14	1.328 (8)	C24—H24	0.9300
N3—C23	1.357 (6)	C25—H25	0.9300
N3—C19	1.329 (9)	C2—C3	1.320 (8)
N4—C1	1.349 (7)	C4—C5	1.504 (9)
N4—C3	1.361 (9)	C4—C6A	1.52 (6)
N5—C1	1.354 (8)	C4—C6	1.536 (19)
N5—C2	1.388 (8)	C4—C7	1.502 (11)
N5—C4	1.510 (6)	C2—H2	0.82 (7)
N4—H4	0.85 (6)	C3—H3	1.02 (7)
N6—C26	1.118 (11)	C5—H5A	0.9600
C8—C9	1.393 (8)	C5—H5B	0.9600
C9—C10	1.355 (9)	C5—H5C	0.9600
C10—C11	1.402 (9)	C6—H6A	0.9600
C11—C12	1.402 (7)	C6—H6B	0.9600
C11—C13	1.418 (8)	C6—H6C	0.9600
C12—C12^i^	1.436 (7)	C6A—H6A2	0.9600
C13—C13^i^	1.344 (10)	C6A—H6A3	0.9500
C14—C15	1.383 (8)	C6A—H6A1	0.9700
C15—C16	1.347 (13)	C7—H7B	0.9600
C16—C17	1.401 (12)	C7—H7C	0.9600
C17—C18	1.417 (7)	C7—H7A	0.9600
C17—C25^i^	1.412 (12)	C26—C27	1.435 (12)
C18—C23^i^	1.431 (10)	C27—H27A	0.9600
C19—C20	1.409 (10)	C27—H27B	0.9600
C20—C21	1.341 (10)	C27—H27C	0.9600
			
N1—Ni1—N2	93.42 (13)	C8—C9—H9	120.00
N1—Ni1—N3	93.77 (18)	C11—C10—H10	120.00
N1—Ni1—N1^i^	79.98 (13)	C9—C10—H10	120.00
N1—Ni1—N2^i^	170.55 (15)	C13^i^—C13—H13	119.00
N1—Ni1—N3^i^	90.91 (18)	C11—C13—H13	119.00
N2—Ni1—N3	96.30 (18)	N2—C14—H14	118.00
N1^i^—Ni1—N2	170.55 (15)	C15—C14—H14	118.00
N2—Ni1—N2^i^	93.90 (13)	C14—C15—H15	121.00
N2—Ni1—N3^i^	79.49 (18)	C16—C15—H15	121.00
N1^i^—Ni1—N3	90.91 (18)	C17—C16—H16	120.00
N2^i^—Ni1—N3	79.49 (18)	C15—C16—H16	119.00
N3—Ni1—N3^i^	173.89 (18)	N3—C19—H19	119.00
N1^i^—Ni1—N2^i^	93.42 (13)	C20—C19—H19	119.00
N1^i^—Ni1—N3^i^	93.77 (18)	C19—C20—H20	120.00
N2^i^—Ni1—N3^i^	96.30 (18)	C21—C20—H20	120.00
Cl1—Ni2—Cl2	133.14 (7)	C20—C21—H21	120.00
Cl1—Ni2—Cl3	104.92 (7)	C22—C21—H21	120.00
Cl1—Ni2—S1	94.21 (6)	C25—C24—H24	119.00
Cl2—Ni2—Cl3	100.47 (6)	C22—C24—H24	119.00
Cl2—Ni2—S1	107.47 (6)	C24—C25—H25	119.00
Cl3—Ni2—S1	118.25 (6)	C17^i^—C25—H25	119.00
Ni2—S1—C1	111.11 (19)	N4—C1—N5	106.5 (6)
Ni1—N1—C8	129.5 (3)	S1—C1—N5	128.4 (4)
Ni1—N1—C12	112.5 (3)	S1—C1—N4	125.1 (5)
C8—N1—C12	118.0 (4)	N5—C2—C3	108.5 (6)
C14—N2—C18	118.0 (4)	N4—C3—C2	107.3 (6)
Ni1—N2—C18	112.9 (4)	N5—C4—C6	110.6 (7)
Ni1—N2—C14	128.9 (4)	N5—C4—C5	109.7 (5)
Ni1—N3—C19	129.3 (4)	C5—C4—C6	104.1 (10)
C19—N3—C23	118.1 (5)	C5—C4—C7	111.4 (6)
Ni1—N3—C23	112.5 (4)	C5—C4—C6A	129 (2)
C1—N4—C3	110.0 (5)	C6—C4—C7	112.5 (10)
C2—N5—C4	124.5 (5)	C6A—C4—C7	93 (2)
C1—N5—C2	107.7 (4)	N5—C4—C7	108.5 (5)
C1—N5—C4	127.7 (5)	N5—C4—C6A	104.0 (19)
C1—N4—H4	121 (5)	N5—C2—H2	118 (4)
C3—N4—H4	128 (5)	C3—C2—H2	133 (4)
N1—C8—C9	122.4 (5)	N4—C3—H3	120 (4)
C8—C9—C10	120.1 (5)	C2—C3—H3	132 (4)
C9—C10—C11	119.5 (5)	C4—C5—H5A	109.00
C10—C11—C12	117.1 (5)	C4—C5—H5B	110.00
C12—C11—C13	119.2 (5)	C4—C5—H5C	110.00
C10—C11—C13	123.7 (5)	H5A—C5—H5B	109.00
N1—C12—C12^i^	117.6 (4)	H5A—C5—H5C	109.00
N1—C12—C11	123.0 (5)	H5B—C5—H5C	109.00
C11—C12—C12^i^	119.5 (4)	C4—C6—H6A	110.00
C11—C13—C13^i^	121.3 (6)	C4—C6—H6B	110.00
N2—C14—C15	123.5 (7)	C4—C6—H6C	109.00
C14—C15—C16	118.6 (8)	H6A—C6—H6B	109.00
C15—C16—C17	121.3 (6)	H6A—C6—H6C	109.00
C16—C17—C18	116.1 (6)	H6B—C6—H6C	109.00
C18—C17—C25^i^	118.7 (6)	C4—C6A—H6A2	110.00
C16—C17—C25^i^	125.2 (5)	C4—C6A—H6A3	110.00
N2—C18—C23^i^	117.6 (4)	H6A1—C6A—H6A2	109.00
N2—C18—C17	122.5 (6)	H6A1—C6A—H6A3	109.00
C17—C18—C23^i^	119.9 (6)	H6A2—C6A—H6A3	110.00
N3—C19—C20	122.1 (5)	C4—C6A—H6A1	109.00
C19—C20—C21	119.5 (7)	H7B—C7—H7C	109.00
C20—C21—C22	120.5 (8)	C4—C7—H7A	109.00
C21—C22—C23	117.0 (6)	C4—C7—H7B	109.00
C21—C22—C24	125.7 (8)	C4—C7—H7C	109.00
C23—C22—C24	117.3 (7)	H7A—C7—H7B	110.00
N3—C23—C18^i^	116.9 (6)	H7A—C7—H7C	110.00
C18^i^—C23—C22	120.3 (5)	N6—C26—C27	179.9 (12)
N3—C23—C22	122.8 (6)	C26—C27—H27A	109.00
C22—C24—C25	122.1 (8)	C26—C27—H27B	110.00
C17^i^—C25—C24	121.6 (6)	C26—C27—H27C	110.00
N1—C8—H8	119.00	H27A—C27—H27B	109.00
C9—C8—H8	119.00	H27A—C27—H27C	110.00
C10—C9—H9	120.00	H27B—C27—H27C	109.00
			
N2—Ni1—N1—C8	8.6 (6)	C1—N5—C2—C3	−0.6 (7)
N3—Ni1—N1—C8	−87.9 (6)	C4—N5—C2—C3	176.5 (5)
N1^i^—Ni1—N1—C8	−178.2 (6)	C2—N5—C1—N4	0.5 (6)
N3^i^—Ni1—N1—C8	88.2 (6)	C2—N5—C4—C7	−117.4 (7)
N2—Ni1—N1—C12	−173.1 (4)	C1—N5—C4—C7	59.2 (8)
N3—Ni1—N1—C12	90.3 (4)	C4—N5—C1—N4	−176.5 (5)
N1^i^—Ni1—N1—C12	0.1 (4)	C2—N5—C1—S1	−177.8 (4)
N3^i^—Ni1—N1—C12	−93.6 (4)	C2—N5—C4—C5	120.8 (7)
N1—Ni1—N2—C14	−92.3 (4)	C1—N5—C4—C6	−176.9 (11)
N3—Ni1—N2—C14	1.9 (4)	C2—N5—C4—C6	6.5 (12)
N2^i^—Ni1—N2—C14	81.7 (4)	N1—C8—C9—C10	0.5 (11)
N3^i^—Ni1—N2—C14	177.4 (4)	C8—C9—C10—C11	−1.8 (11)
N1—Ni1—N2—C18	84.0 (3)	C9—C10—C11—C12	1.4 (11)
N3—Ni1—N2—C18	178.2 (3)	C9—C10—C11—C13	−179.0 (7)
N2^i^—Ni1—N2—C18	−102.0 (3)	C12—C11—C13—C13^i^	−2.3 (11)
N3^i^—Ni1—N2—C18	−6.3 (3)	C10—C11—C12—N1	0.2 (10)
N1—Ni1—N3—C19	9.7 (5)	C13—C11—C12—C12^i^	0.9 (10)
N2—Ni1—N3—C19	−84.2 (5)	C10—C11—C12—C12^i^	−179.6 (6)
N1^i^—Ni1—N3—C19	89.7 (5)	C13—C11—C12—N1	−179.4 (6)
N2^i^—Ni1—N3—C19	−177.0 (5)	C10—C11—C13—C13^i^	178.1 (8)
N1—Ni1—N3—C23	−167.3 (3)	N1—C12—C12^i^—C11^i^	−180.0 (6)
N2—Ni1—N3—C23	98.9 (3)	C11—C12—C12^i^—N1^i^	−180.0 (6)
N1^i^—Ni1—N3—C23	−87.3 (3)	N1—C12—C12^i^—N1^i^	0.3 (8)
N2^i^—Ni1—N3—C23	6.1 (3)	C11—C12—C12^i^—C11^i^	−0.2 (9)
Cl3—Ni2—S1—C1	−81.0 (2)	C11—C13—C13^i^—C11^i^	3.1 (12)
Cl1—Ni2—S1—C1	169.56 (19)	N2—C14—C15—C16	−2.6 (9)
Cl2—Ni2—S1—C1	31.7 (2)	C14—C15—C16—C17	2.5 (10)
Ni2—S1—C1—N4	−3.1 (5)	C15—C16—C17—C25^i^	178.2 (6)
Ni2—S1—C1—N5	175.0 (4)	C15—C16—C17—C18	−0.4 (9)
Ni1—N1—C8—C9	179.3 (5)	C18—C17—C25^i^—C24^i^	1.8 (9)
C8—N1—C12—C12^i^	178.3 (6)	C16—C17—C25^i^—C24^i^	−176.8 (7)
Ni1—N1—C12—C11	−180.0 (5)	C16—C17—C18—C23^i^	177.6 (5)
C12—N1—C8—C9	1.1 (10)	C25^i^—C17—C18—N2	179.4 (5)
Ni1—N1—C12—C12^i^	−0.2 (6)	C25^i^—C17—C18—C23^i^	−1.1 (8)
C8—N1—C12—C11	−1.5 (9)	C16—C17—C18—N2	−1.9 (8)
Ni1—N2—C18—C23^i^	5.6 (5)	C17—C18—C23^i^—C22^i^	−0.5 (8)
C14—N2—C18—C17	2.0 (7)	N2—C18—C23^i^—N3^i^	−0.4 (7)
Ni1—N2—C18—C17	−174.8 (4)	C17—C18—C23^i^—N3^i^	−179.9 (5)
Ni1—N2—C14—C15	176.6 (4)	N2—C18—C23^i^—C22^i^	179.0 (5)
C14—N2—C18—C23^i^	−177.6 (4)	N3—C19—C20—C21	0.4 (10)
C18—N2—C14—C15	0.4 (7)	C19—C20—C21—C22	−1.8 (10)
Ni1—N3—C19—C20	−175.5 (4)	C20—C21—C22—C24	−179.1 (7)
C23—N3—C19—C20	1.3 (8)	C20—C21—C22—C23	1.4 (10)
C19—N3—C23—C22	−1.7 (8)	C24—C22—C23—C18^i^	1.5 (8)
Ni1—N3—C23—C18^i^	−5.0 (6)	C21—C22—C23—N3	0.4 (9)
C19—N3—C23—C18^i^	177.6 (5)	C24—C22—C23—N3	−179.2 (5)
Ni1—N3—C23—C22	175.6 (4)	C23—C22—C24—C25	−0.9 (10)
C3—N4—C1—N5	−0.3 (6)	C21—C22—C24—C25	179.6 (7)
C3—N4—C1—S1	178.2 (4)	C21—C22—C23—C18^i^	−179.0 (6)
C1—N4—C3—C2	−0.1 (7)	C22—C24—C25—C17^i^	−0.8 (11)
C4—N5—C1—S1	5.2 (8)	N5—C2—C3—N4	0.5 (7)
C1—N5—C4—C5	−62.7 (8)		

Symmetry codes: (i) −*x*, *y*, −*z*+1/2.

### Hydrogen-bond geometry (Å, °)

**Table:**

*D*—H···*A*	*D*—H	H···*A*	*D*···*A*	*D*—H···*A*
N4—H4···Cl2	0.85 (6)	2.37 (7)	3.178 (6)	160 (6)
C2—H2···Cl3^ii^	0.82 (7)	2.77 (7)	3.552 (8)	160 (4)
C5—H5C···S1	0.96	2.75	3.402 (9)	126
C7—H7A···S1	0.96	2.68	3.409 (8)	133
C10—H10···Cl3^iii^	0.93	2.72	3.557 (7)	151
C25—H25···N6^iv^	0.93	2.60	3.502 (9)	162

Symmetry codes: (ii) −*x*+1, −*y*+2, −*z*+1; (iii) −*x*+1/2, −*y*+3/2, −*z*+1; (iv) *x*−1/2, −*y*+1/2, *z*−1/2.
